# Effects of Platelet-Rich Fibrin on In Vitro Periodontal Ligament Cell Functions

**DOI:** 10.3390/biomedicines13102360

**Published:** 2025-09-26

**Authors:** Pablo Cores Ziskoven, Andressa Vilas Boas Nogueira, Jean-Claude Imber, Philipp Bani, Charlott Luise Hell, Jens Weusmann, James Deschner

**Affiliations:** 1 Department of Periodontology and Operative Dentistry, University Medical Center, Johannes Gutenberg University, 55131 Mainz, Germany; a.nogueira@uni-mainz.de (A.V.B.N.); naufalphilipp.bani@unimedizin-mainz.de (P.B.); charlottluise.hell@unimedizin-mainz.de (C.L.H.); jens.weusmann@unimedizin-mainz.de (J.W.); james.deschner@uni-mainz.de (J.D.); 2 Department of Periodontology, School of Dental Medicine, University of Bern, 3010 Bern, Switzerland; jean-claude.imber@unibe.ch

**Keywords:** platelet-rich fibrin, periodontal ligament cells, wound healing, periodontitis

## Abstract

**Background:** Periodontitis is a chronic inflammatory disease that leads to tooth loosening and ultimately tooth loss. Regenerative approaches employing bioactive substances aim to restore lost tissues. Platelet-rich fibrin (PRF) is a simple and cost-effective option, but its effects on periodontal ligament (PDL) cells under inflammatory conditions remain unclear. **Objectives:** This study investigated the stimulating effects of platelet-rich fibrin on molecules crucial for periodontal wound healing and tissue remodelling in periodontal ligament (PDL) cells, under normal and inflammatory conditions mimicked by TNF-α. **Methods** The stimulating effects of different concentrations of PRF on the gene expression of VEGF, BMP2, COX2, TNF-α, and SPP1 were analysed by real-time PCR and ELISA. In addition, the possible modulating effects of TNF-α, a pro-inflammatory cytokine associated with periodontitis, on PRF-induced effects were studied. Furthermore, cell viability, proliferation, and migration were investigated. **Results**: A 2–3-fold dose-dependent increase in the expression of all the aforementioned genes by PRF was observed at 24 h and 48 h. Additional incubation with TNF-α did not lead to any significant modulation of PRF-induced expression patterns, indicating that the effects of PRF were not compromised in an inflammatory environment. Functionally, PRF caused a significant 35% increase in cell migration between 24 h and 48 h, which was again not affected by a pro-inflammatory condition. Cell viability and proliferation remained largely unaffected by PRF, irrespective of the presence of TNF-α or not. **Conclusions**: The results suggest that PRF can promote initial periodontal wound healing even in an inflammatory environment by stimulating the expression of cytokines, growth factors and markers of osteogenic differentiation such as VEGF, BMP2 and SPP1, which are involved in angiogenesis, tissue remodelling, and/or cell migration.

## 1. Introduction

Periodontitis is characterized by host-induced irreversible destruction of the periodontium and, if left untreated, leads to periodontal pockets and bone destruction, tooth loosening, tooth loss, and thus to functional and aesthetic deficits [1]. According to the stepwise treatment guidelines of the European Federation of Periodontology [2,3], all local and systemic etiologic factors should be identified and, if possible, treated in the first stage of periodontitis therapy in order to enable the success of the therapy in the second stage, i.e., the non-surgical removal of pathogenic biofilms from the root surfaces. If periodontal signs of inflammation such as deep probing depths and bleeding on probing still exist after evaluation of the findings, surgery such as resective and/or regenerative techniques can be applied. This third stage of systematic periodontal therapy is of crucial importance if the first two stages of therapy have not led to the desired results, as it has been shown that untreated residual periodontal pockets increase the risk of tooth loss [4,5]. In contrast to reparative or resective procedures, regenerative procedures aim to restore all tissues of the periodontium and thus generate a genuine new attachment between the alveolar bone and the root surface [6]. It has been shown that the regenerative potential comes primarily from periodontal ligament (PDL) cells [7]. Biologically active agents such as enamel matrix proteins modulate the cellular regeneration processes and are well studied and established [8,9,10,11,12,13]. However, the handling and application of these preparations is technically sensitive and relatively expensive. Since the beginning of this millennium, autologous blood concentrates have therefore been increasingly investigated and established as bioactive signalling substances for periodontal regeneration as alternative preparations [14,15,16]. Platelet-rich fibrin (PRF), as a prominent representative of this group, combines several advantages: mechanical stability in the sense of a resilient scaffold for the immigration of various cell types, a significantly potentiated output of biologically active molecules, simple handling and low costs [17]. The autologous fibrin matrix has high angiogenic potential, is capable of recruiting circulating stem cells and, thanks to its relatively stable three-dimensional structure, enables an environment conducive to wound healing [18,19,20,21,22,23,24,25,26]. Surprisingly, despite the relatively high number of clinical studies, there are only a few in vitro trials and histological studies on the use of PRF in an inflammatory environment. This study sought to examine the stimulating effects of PRF on PDL cell functions critical in wound healing and tissue remodelling, under both normal and inflammatory conditions.

## 2. Materials and Methods

### 2.1. Preparation of PRF

To prepare the PRF [27], venous blood was obtained from a healthy, male, adult person with a generally normal medical history, after written informed consent. The procedure was approved by the Ethics Committee of the Medical Association of Rhineland-Palatinate, Mainz, Germany (approval no. 14821_1). Blood was always taken in the morning and on an empty stomach. A tourniquet (Medi-Inn venous tourniquet, 42–45 cm, Hirten, Germany) was placed on the right or left arm at the level of the biceps brachii muscle with controlled pressure. After thorough disinfection (Softasept N, Braun, Melsungen, Germany) of the cubital fossa, blood was drawn preferably from the median cubital vein. A butterfly system (safety blood collection set, Greiner Bio-One, Frickenhausen, Germany) and uncoated tubes (Zhejiang Gongdong Medical Technology, Taizhou, China) were used for this purpose. Ten ml of blood was collected per tube, which is sufficient for the production of one PRF clot. For centrifugation, tubes were always placed in pairs; if only one blood tube was available, a counterbalance tube filled with sterile water of equal weight was used. The tubes were transferred to the centrifuge (PRF DUO Quattro, Mectron, Cologne, Germany) provided for this purpose and centrifuged at 1300 rpm for 8 min. The fibrin portion was then removed from the tube, using sterile forceps and scissors, and separated from the reddish corpuscular erythrocyte-rich portion. The fibrin clots were transferred to a sterilized stainless steel box, compressed with light, standardized pressure and transported to the local cell laboratory. Under aseptic conditions, the prepared fibrin clots were transferred to prepared 6-well plates. These contained 3 mL of Dulbecco’s Modified Eagle Medium (DMEM, Invitrogen, Thermo Fisher Scientific, Waltham, MA, USA) enriched with 10% fetal bovine serum (FBS, Invitrogen) as well as 100 U/mL penicillin (Invitrogen) and 100 μg/mL streptomycin (Invitrogen). The loaded plate was stored for 48 h in an incubator at 37 °C in a humidified atmosphere of 5% CO_2_. After 24 h, the medium containing the soluble factors of the clot was then collected from each well and used as PRF for the further experiments.

### 2.2. Cell Culture

A PDL cell line from the University of Göttingen was used for the cell culture, as in our previous experiments [28]. The cell line was obtained from the third molar of a healthy 26-year-old male non-smoker after obtaining written consent according to the ethics regulations of the University of Goettingen (approval no.: 27/2/09). For seeding, the cells were removed from the storage in liquid nitrogen, thawed and transferred to cell culture flasks with culture medium (DMEM GlutaMAX, Invitrogen) enriched with 10% FBS. Furthermore, 100 U/mL penicillin and 100 μg/mL streptomycin were added. The bottle filled with cells and medium was then placed horizontally in a cell incubator. The conditions in the incubator were 37 °C in a humidified atmosphere of 5% CO_2_. To check the growth of the cells, they were regularly examined under a light microscope and were grown until confluence of 70–80%. Then, cells were seeded in 6-well plates (100,000 cells/well) at a volume of 2 mL. The medium was changed every second day and the cells were checked using optical magnification. One day before treatment, the FBS concentration was reduced to 1%. To simulate an inflammatory environment in vitro, PDL cells were treated with TNF-α (recombinant human TNF-α protein, Cat.# 300-01A, PeproTech, Thermo Fisher Scientific) [29]. For dose-dependent experiments, TNF-α was used at concentrations of 5 ng/mL for 24 h. To investigate whether PRF had a dose-dependent effect on the PDL cells, the cells were incubated with different PRF concentrations: 100% (undiluted), 50%, and 25% for 24 h. To test, if PRF-stimulated effects were modulated by an inflammatory environment, cells were exposed to PRF 100% and/or TNF-α (5 ng/mL) for 24 h and 48 h. All experiments were performed three times.

### 2.3. Real-Time PCR

RNA extraction was performed using the RNeasy Mini Kit (Qiagen, Hilden, Germany) according to the manufacturer’s instructions. NanoDrop ND-2000 (Thermo Fischer Scientific) spectrophotometer was used in order to determine the RNA concentration. Five hundred ng of total RNA was reverse transcribed using the iScrip Select cDNA Synthesis Kit (Bio-Rad Laboratories, Munich, Germany) according to the manufacturer’s protocol. Gene expression analysis of vascular endothelial growth factor (VEGF), bone morphogenetic protein 2 (BMP2), cyclooxygenase 2 (COX-2), tumor necrosis factor alpha (TNF-α), osteopontin (SPP1), and glyceraldehyde 3-phosphate dehydrogenase (GAPDH) was performed using the PCR thermal cycler CFX96 (Bio-Rad Laboratories), SYBR green PCR master mix (QuantiFast SYBR Green PCR Kit, Qiagen), and specific primers (QuantiTect Primer Assay, Qiagen). One µL of cDNA was mixed with 12.5 µL master mix, 2.5 µL primer, and 9 µL nuclease-free water. The mix was heated at 95 °C for 5 min, followed by 40 cycles of denaturation at 95 °C for 10 s, and a combined annealing/extension steps at 60 °C for 30 s. Data were analyzed by the comparative threshold cycle method.

### 2.4. ELISA

The protein levels of VEGF and TNF-α in the cell supernatants at 24 h and 48 h were measured using commercially available ELISA kits (DuoSet, R&D Systems, Minneapolis, MN, USA) according to the manufacturer’s instructions. The optical density was determined using a microplate reader (BioTek Synergy H1, Agilent, Santa Clara, CA, USA) set to 450 nm. The readings at 450 nm were subtracted from the readings at 540 nm for optical correction as per manufacturer’s recommendation. The cell numbers were checked at the end of the experiments and were found to be not significantly different between the groups.

### 2.5. In Vitro Wound Healing and Cell Migration

To analyze the cellular migration, PDL cells were seeded at a concentration of 2.0 × 104 cells in special cell migration culture plates with a silicone insert separating the well into two compartments (Culture-Insert 2 Well, µ-Dish 35 mm, ibiTreat, Cat.# 81176, Ibidi, Gräfelfing, Germany). Seventy μL of the cell suspension was added to each compartment according to the manufacturer’s instructions and incubated for one day at 37 °C and 5% CO_2_ in the incubator. After 24 h and removal of the insert, all non-adherent cells were removed by several washing steps with phosphate-buffered saline (PBS). Afterwards, the monolayers with the cell-free areas were again incubated with medium containing 1% FBS. The monolayers were then treated with PRF 100% and/or TNF-α (5 ng/mL) for up to 48 h. Untreated monolayers served as control. The defined cell free area in the cell layer was photographed every 30 min for 48 h. Wound closure was documented and analyzed over time using a JuLI^TM^ Br and the JuLI^TM^ Br PC software (Ver 1.3.7.5, both NanoEnTek, Seoul, Republic of Korea). For data analysis, the size of the cell-free area was automatically determined using the freely available Image J program (Ver 1.54m) and the “MRI_Wound_Healing_Tool” plug-in. Each experiment was performed three times.

### 2.6. Cell Viability

To test whether PRF and/or TNF-α have a cytotoxic effect on the cells, the LIVE/DEAD Viability/Cytotoxicity Kit (Cat.# L3224, Molecular Probes, Invitrogen) and fluorescence microscopy were applied. The principle of this assay is based on the esterase activity in living cells. These enzymes can cleave calcein to calcein-AM, which fluoresces green at a wavelength of λ = ~495/~515 nm. Dead cells can be detected using ethidium homodimer (EthD-1), which infiltrates cells with defective walls, fluoresces red at a wavelength of λ = ~495/~635 nm and, thereby, marks them as dead. Cells were cultured in 24-well plates with a density of 20,000 cells/well according to the protocol described. At 70–80% confluence, FBS was reduced to 1% and cells were treated for 24 h and the following day for 48 h according to the experimental design. For fluorescence microscopy, the staining solutions (2 μM calcein and 4 μM EthD-1) were mixed by vortexing with 10 mL sterile PBS. After the cells were washed twice with PBS, 500 μL of the staining solution were added. Fluorescence microscopy was performed using ZOE^TM^ (Fluorescent Cell Imager, Bio-Rad Laboratories). Each experiment was performed three times.

### 2.7. Cell Proliferation

Cells were seeded in 96-well plates at a density of 5000 cells/well and grown for 24 h. Then, FBS was reduced to 1% for another 24 h. Afterwards, cells were treated with PRF in the presence and absence of TNF-α. Untreated cells served as control. After 24 h and 48 h, the WST-1 assay was performed to measure the cell proliferation according to the manufacturer’s instructions (Roche, Basel, Switzerland). Briefly, after incubation of the cells with the dye at 37 °C and 5% CO_2_ for 4 h, the absorbance was determined at 450 nm and 640 nm as reference wavelength.

### 2.8. Statistical Analysis

First, the result values were tested graphically and mathematically for normal distribution using the Shapiro–Wilk test and QQ plot (quantile–quantile plot), with a significance level of α = 0.05. Parametric tests were then applied for normally distributed data sets and non-parametric tests for non-normally distributed data. An analysis of variance (one-way ANOVA) was used to analyse the mean values of normally distributed groups, while a Kruskal–Wallis test was applied in the case of non-normal distribution. Data identified as normally distributed and classified as significantly different were further examined with post hoc tests (Dunnett). Non-parametric post hoc tests (Dunn) were used for non-normally distributed data. All experiments were performed in triplicate and repeated twice at different time points.

## 3. Results

### 3.1. Dose-Dependent Effects of PRF on the VEGF, BMP2, TNF-α, COX2 and SPP1 Gene Expression in PDL Cells

**Figure 1:**
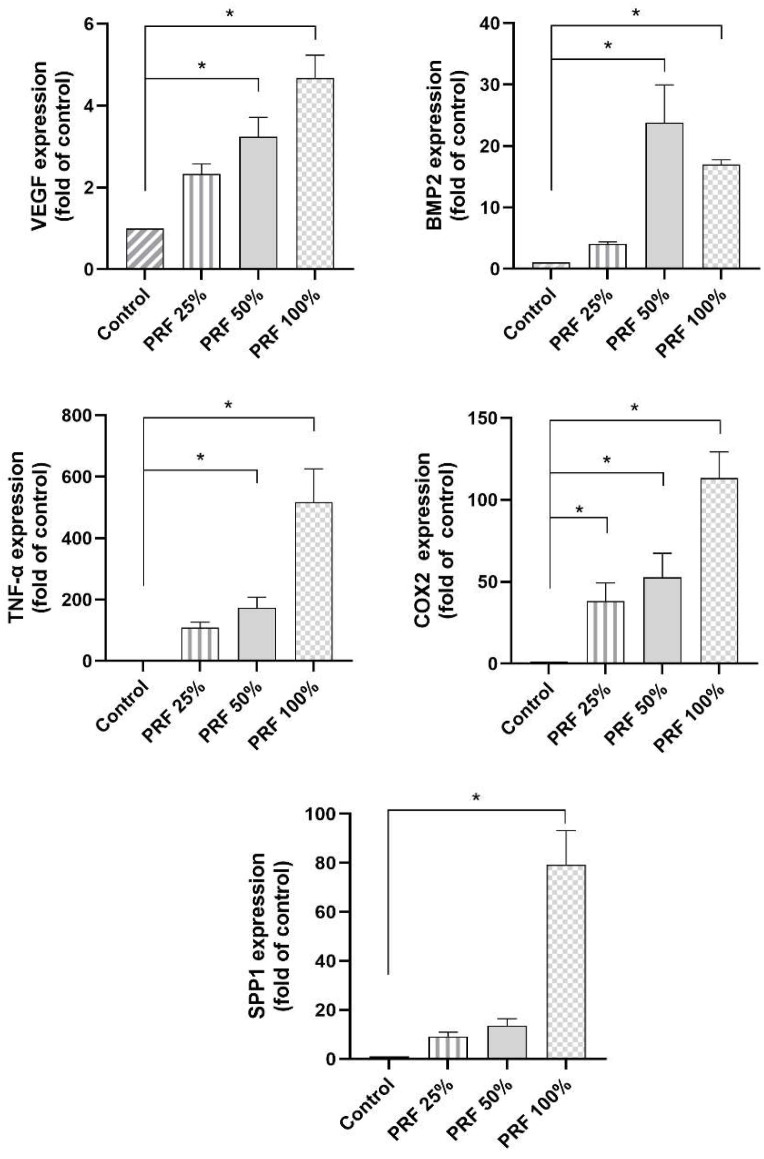
Effects of different concentrations (25%, 50%, 100%) of PRF on the VEGF, BMP2, TNF-α, COX2 and SPP1 gene expression at 24 h. Untreated cells served as control. * Significant (*p* < 0.05) difference between groups.

First, we studied if PRF had a dose-dependent effect on the gene expression of molecules, which are involved in periodontal tissue healing after 24 h. A significant increase in the expression of these molecules, depending on the PRF concentration, was observed (Figure 1). PRF 100% led to a statistically significant increase in the expression of all the studied genes as compared to control (Figure 1). PRF 50% also caused significant expression changes for most markers, except for SPP1 (Figure 1). By contrast, PRF 25% only led to a significant upregulation of the COX2 gene expression (Figure 1).

### 3.2. PRF-Induced VEGF, BMP2, COX2, TNF-α and SPP1 Gene Expression Under Normal and Inflammatory Conditions

**Figure 2:**
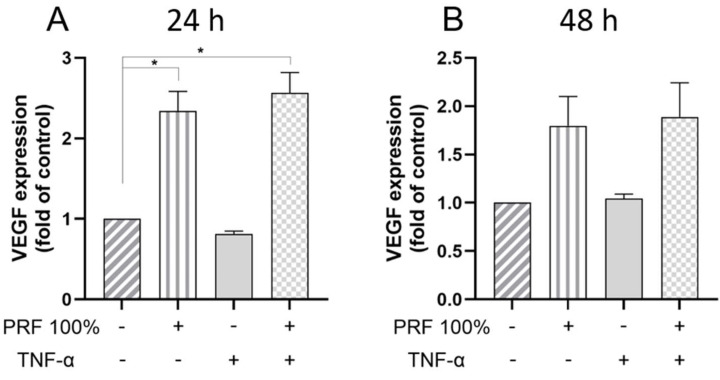
Effects of PRF (100%) and/or TNF-α (5 ng/mL) on the VEGF gene expression at 24 h (**A**) and 48 h (**B**). Untreated cells served as control. * Significant (*p* < 0.05) difference between groups.

**Figure 3:**
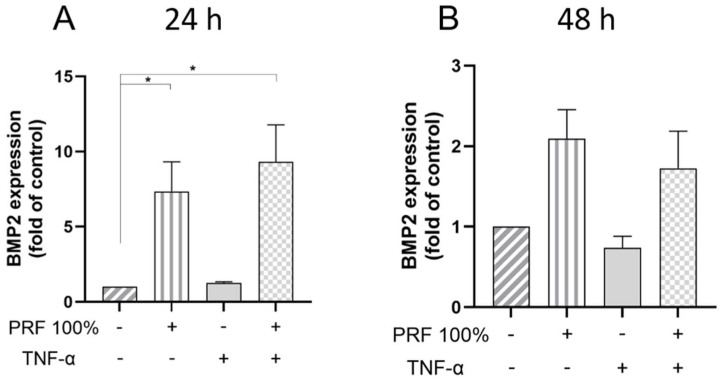
Effects of PRF (100%) and/or TNF-α (5 ng/mL) on the BMP2 gene expression at 24 h (**A**) and 48 h (**B**). Untreated cells served as control. * Significant (*p* < 0.05) difference between groups.

**Figure 4:**
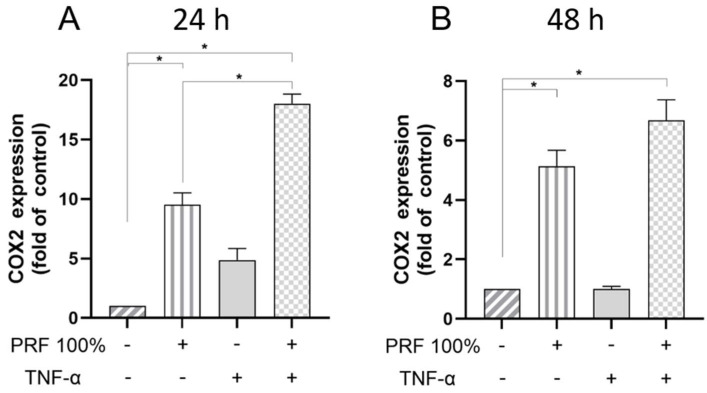
Effects of PRF (100%) and/or TNF-α (5 ng/mL) on the COX2 gene expression at 24 h (**A**) and 48 h (**B**). Untreated cells served as control. * Significant (*p* < 0.05) difference between groups.

**Figure 5:**
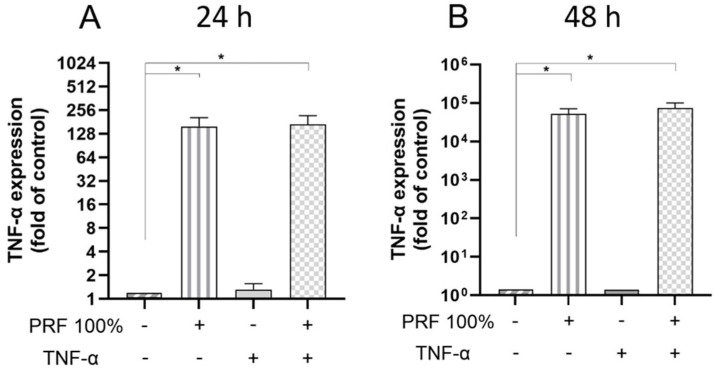
Effects of PRF (100%) and/or TNF-α (5 ng/mL) on the TNF-α gene expression at 24 h (**A**) and 48 h (**B**). Untreated cells served as control. * Significant (*p* < 0.05) difference between groups.

**Figure 6:**
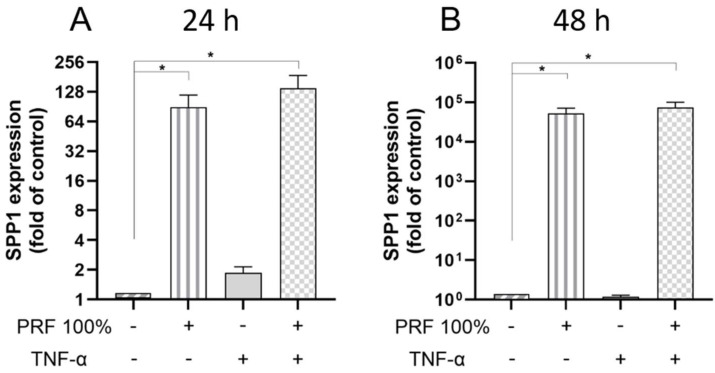
Effects of PRF (100%) and/or TNF-α (5 ng/mL) on the SPP1 gene expression at 24 h (**A**) and 48 h (**B**). Untreated cells served as control. * Significant (*p* < 0.05) difference between groups.

Furthermore, we analysed whether the stimulatory effects of PRF were modulated by TNF-α, which is a key mediator in both inflammation and initial wound healing (Figure 2, Figure 3, Figure 4, Figure 5 and Figure 6). The VEGF expression was again upregulated by PRF 100%, which was significant at 24 h but not at 48 h (Figure 2). The same cell response pattern to PRF was observed under inflammatory conditions mimicked by TNF-α at both time points. The PRF-induced VEGF gene expression did not significantly differ between normal and inflammatory conditions. Similarly to VEGF, BMP2 was significantly upregulated by PRF in the presence and absence of TNF-α at 24 h (Figure 3). In addition, PRF had a stimulatory but not significant impact on the BMP2 expression at 48 h under both normal and inflammatory conditions (Figure 3). The BMP2 gene expression upregulated by PRF did not differ significantly between normal and inflammatory condition (Figure 3).

Moreover, PRF caused a significant upregulation of the COX2 and TNF-α gene expression in a normal and inflammatory environment at 24 h and 48 h (Figure 4 and Figure 5). The PRF-induced COX2 and TNF-α gene expression did not significantly differ between normal and inflammatory conditions except for COX2 at 24 h, where the PRF-stimulated COX2 expression was further and significantly increased under inflammatory condition (Figure 4 and Figure 5).

Finally, we examined the actions of PRF on SPP1 in the presence and absence of TNF-α. Again, PRF caused a significant upregulation of the SPP1 gene expression, which was observed under normal and inflammatory conditions at 24 h and 48 h (Figure 6). The PRF-induced SPP1 gene expression did not significantly differ between normal and inflammatory conditions (Figure 6).

### 3.3. PRF-Induced VEGF and TNF-α Protein Levels Under Normal and Inflammatory Conditions

**Figure 7:**
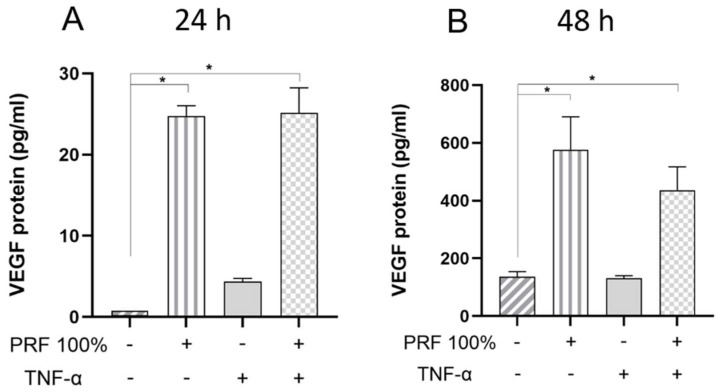
Effects of PRF (100%) and/or TNF-α (5 ng/mL) on the VEGF protein levels in the supernatants at 24 h (**A**) and 48 h (**B**). Untreated cells served as control. * Significant (*p* < 0.05) difference between groups.

**Figure 8:**
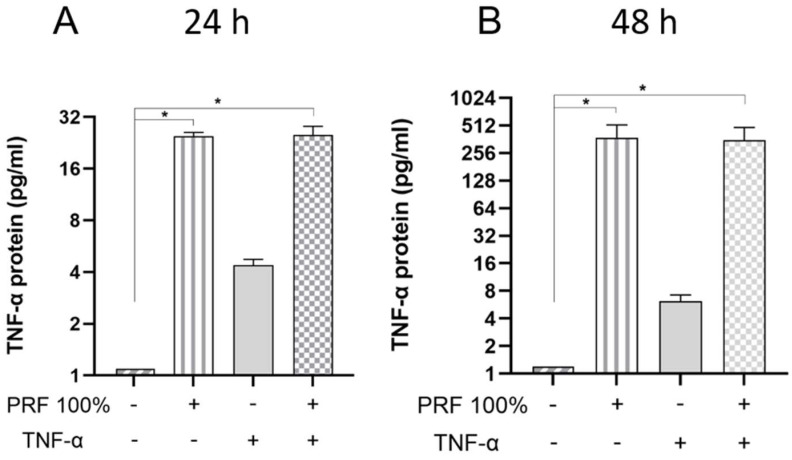
Effects of PRF (100%) and/or TNF-α (5 ng/mL) on the TNF-α protein levels in the supernatants at 24 h (**A**) and 48 h (**B**). Untreated cells served as control. * Significant (*p* < 0.05) difference between groups.

We also investigated whether the effects of PRF on PDL cells can also be found at the protein level. As measured in the supernatants by ELISA, PRF led to a significant increase in VEGF and TNF-α protein under normal and inflammatory conditions at 24 h and 48 h (Figure 7 and Figure 8). Again, no significant difference was observed for the VEGF and TNF-α protein levels between normal and inflammatory settings (Figure 7 and Figure 8).

### 3.4. Effects of PRF on In Vitro Wound Closure Under Normal and Inflammatory Conditions

**Figure 9:**
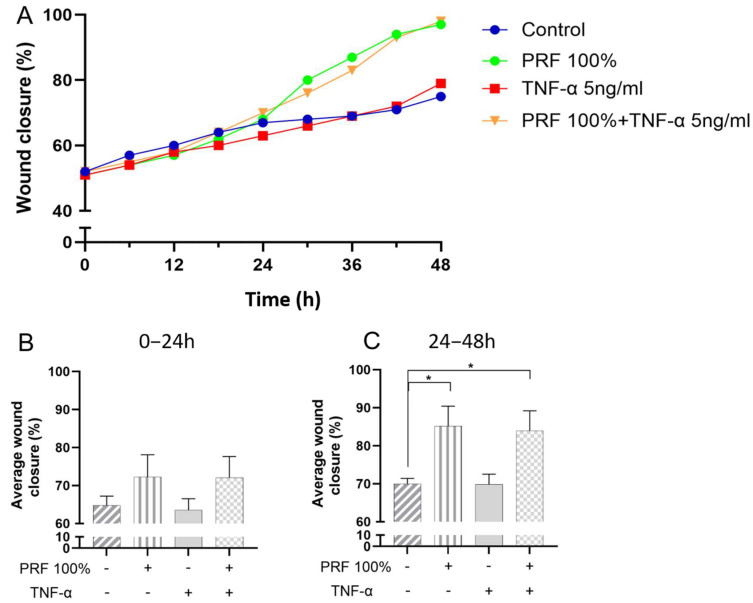
(**A**) Effects of PRF (100%) and/or TNF-α (5 ng/mL) on the in vitro wound closure over 48 h. (**B**) Average in vitro wound closure (%), as shown in A, for the first 24 h. (**C**) Average in vitro wound closure (%), as shown in A, for the last 24 h. Untreated cells served as control. * Significant (*p* < 0.05) difference between groups.

PDL cell monolayers with cell-free areas were incubated with PRF in presence or absence of TNF-α. Closure of the cell-free areas were studied over a period of 48 h. The average wound closure across all four groups exhibited a similar steady progression within the first 24 h (Figure 9A,B). However, after 24 h, the wound closure was significantly more enhanced in the monolayers which were exposed to PRF 100% and PRF 100%+TNF-α as compared to control (Figure 9C). No difference was observed between PRF-induced wound closure under normal and inflammatory conditions.

### 3.5. Effects of PRF on PDL Cell Viability Under Normal and Inflammatory Conditions

**Figure 10:**
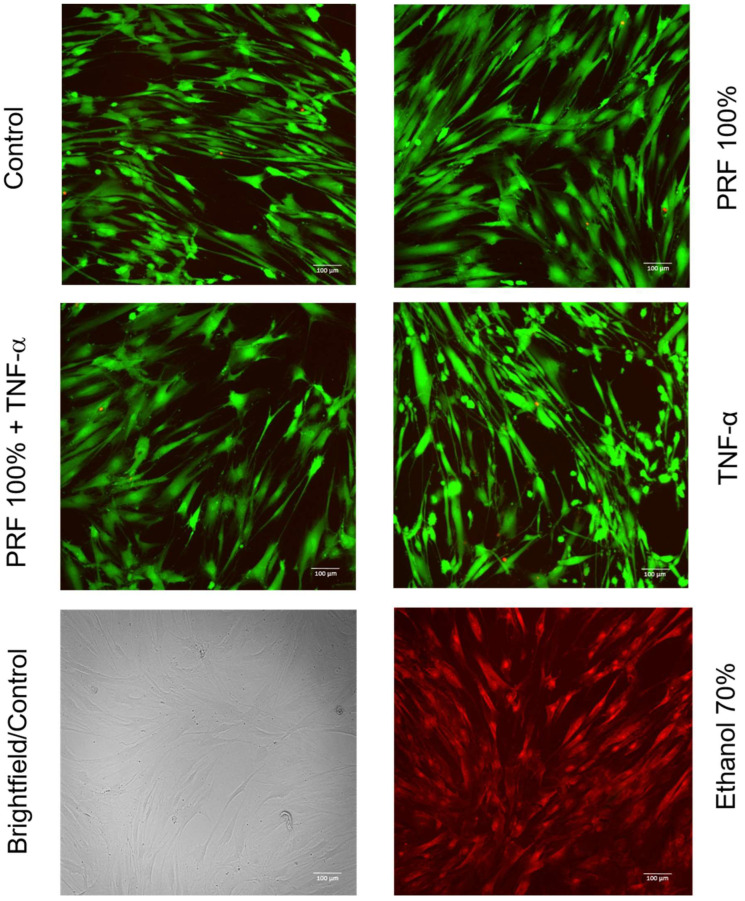
Effects of PRF (100%) and/or TNF-α (5 ng/mL) on the PDL cell viability at 48 h. Representative images from three independent experiments are shown.

As shown in Figure 10, cell viability and morphology were not altered in PDL cells exposed to PRF 100% and/or TNF-α as compared to control at 48 h.

### 3.6. Effects of PRF on Cell Proliferation of PDL Cells Under Normal and Inflammatory Conditions

**Figure 11:**
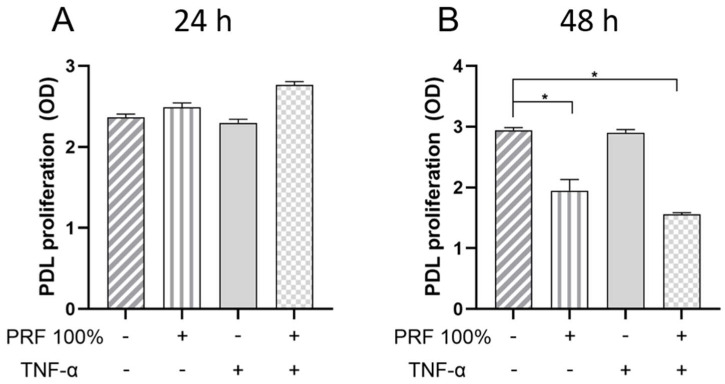
Effects of PRF (100%) and/or TNF-α (5 ng/mL) on the PDL cell proliferation at 24 h. (**A**) and 48 h (**B**). Untreated cells served as control. * Significant (*p* < 0.05) difference between groups.

At 24 h, no significant effect of PRF and/or TNF-α on cell proliferation was observed (Figure 11). However, at 48 h, TNF-α caused significant inhibition of cell proliferation in both the presence and absence of PRF (see Figure 11).

## 4. Discussion

Our study investigated the effects of PRF on markers that are crucial for periodontal wound healing and tissue remodelling in PDL cells under normal and inflammatory conditions. PRF induced upregulation of VEGF, BMP2, COX2, TNF-α, and SPP1 and also stimulated wound closure in vitro. A proinflammatory challenge had no impact on the actions of PRF, suggesting that even in an inflammatory environment, the beneficial effects of PRF may be fully preserved.

In our experiments, PRF was found to increase VEGF, BMP2, COX2, TNF-α, and SPP1 in a dose-dependent manner, highlighting its beneficial role in soft and hard tissue formation and wound healing. Our findings are consistent with the results of other studies that have demonstrated the stimulating effects of PRF on these molecules [25,30]. Autologous PRF contains platelets and leukocytes embedded in a stable fibrin matrix, as well as growth and differentiation factors that exert pleiotropic biological effects [18,24,25,31,32]. These factors can directly and indirectly promote the proliferation, migration, and differentiation of soft and hard tissue cells, including fibroblasts, endothelial cells, osteoblasts, and chondrocytes [32,33]. To investigate the effects of PRF in our experiments, PRF was prepared according to a well-established protocol [27,34].

VEGF is a growth factor produced by various cell types and with critical impact on wound healing by its effects on angiogenesis, vasodilation, remodelling as well as cell migration and proliferation. BMPs are multifunctional growth factors of the transforming growth factor beta superfamily and play a decisive role in bone development, homeostasis and healing. They have been shown to induce ectopic new bone formation in animal models [35,36].

Interestingly, our experiments showed that PRF led to a significant increase in the gene expression and protein synthesis of TNF-α. This effect of PRF could be beneficial in the context of initial wound healing, as TNF-α attracts immunocompetent cells such as neutrophil granulocytes, which are responsible for the removal of wound debris and the resorption and remodelling of the blood coagulum into a more stable network [37,38]. The importance of the physiological role of TNF-α in the process of early wound healing was also demonstrated in animal models: Here it was shown that there was a sharp increase in TNF-α synthesis immediately after mechanical trauma to the skin, with its zenith after 24 h to 48 h [39]. Afterwards, the expression decreased to basal levels. In addition, it was shown that treating the animals with anti-TNF-α monoclonal antibody led to significantly reduced fibroblast and granulocyte numbers and thus resulted in significantly slower wound closure overall. On the other hand, the application of exogenous TNF-α also led to accelerated wound closure. This underlines the importance of a controlled high TNF-α concentration in the initial phase of wound healing, which was stimulated by PRF in our study. TNF-α plays a dual role in wound healing. While persistently elevated TNF-α levels contribute to inflammation, tissue degradation, and impaired regeneration, low or transient concentrations can support the healing process by stimulating angiogenesis, fibroblast activity, and extracellular matrix turnover. This ambivalent role may explain why, in our experimental setting, the addition of TNF-α did not abolish the stimulatory effects of PRF on periodontal ligament cells.

An early increase after mechanical trauma was also observed for COX2 in an animal study [40]. Analogous to TNF-α, selective COX2 inhibitors also led to slower re-epithelialization and inhibited angiogenesis. Our study also revealed that PRF induced the increase in COX2, which might be beneficial in early wound healing. With regard to the controlled and necessary inflammation in the initial phase of wound healing, the PRF-mediated upregulation of TNF-α and COX-2 may therefore be considered beneficial. It is known that TNF-α can exert regulatory effects on growth and differentiation of many cell types, indicating that the effects of TNF-α on tissue formation are indirect [41]. For example, TNF-α has been shown to promote VEGF expression, which induces wound revascularization and angiogenesis under controlled, short-term exposure, as found in wound healing [42,43,44].

Another protein investigated in our study is osteopontin (SPP1), which, due to its early isolation from bone tissue, was thought to be mainly involved in bone metabolism [45]. However, the gene coding for SPP1 was soon identified as identical to a gene responsible for cell differentiation. It is now known that SPP1 is widely distributed and also has cytokine functions in other cells and tissues such as macrophages and is involved in wound healing [46]. In vitro studies have shown that SPP1 can act as a migration stimulus, have regulatory effects on gene expression and cell differentiation of various cells [47] and enable the attraction of immunocompetent cells. The presence of SPP1 in wound areas, especially in connection with leukocytes, has been documented in the literature [48,49]. Mice lacking a functional SPP1 gene show negatively altered wound healing properties [46]. Our experiments showed that PRF led to a dose-dependent increase in SPP1 expression, suggesting that the beneficial actions of PRF might be mediated also through this molecule. The absence of a significant increase in SPP1 expression in the PRF 25% and PRF 50% groups could indicate that a threshold concentration is required to trigger a measurable induction of this molecule, which was only achieved at a concentration of PRF 100%. In addition, a possible positive feedback mechanism may only become effective at higher concentrations, which could explain the stronger induction at PRF 100%.

Since our experiments revealed that PRF increases the synthesis of growth and differentiation factors, which in turn have a stimulating effect on wound healing, we also investigated the influence of PRF on wound closure in vitro in further experiments. These showed that PRF indeed enhances wound healing, which again underscores the pro-healing effect of PRF. Our observation is in accordance with studies on periodontal cells by other investigators [27,50].

Our experiments demonstrated that PRF did not alter the viability and morphology of PDL cells. Likewise, no effect of PRF on cell proliferation was observed at 24 h. Interestingly, cell proliferation at 48 h was lower in PRF-treated cell cultures than in the control. Further studies are needed to clarify whether PRF may have promoted the differentiation of PDL cells at the expense of proliferation after the initial 24 h.

PRF is often used clinically at sites where inflammation has not yet been completely resolved. Furthermore, PRF is mainly applied in combination with surgical procedures that lead to traumatic inflammation. In addition, the first phase of wound healing involves inflammatory processes. A unique aspect of our study is that the stimulatory effects of PRF were also investigated in an inflammatory environment. While most previous in vitro studies examined PRF only under normal conditions, its clinical application usually takes place in sites where inflammation is still present. To the best of our knowledge, however, there are few in vitro studies to date that have investigated whether the stimulating effects induced by PRF are also maintained in an inflammatory environment. We therefore performed additional experiments to investigate whether the effects of PRF are altered under inflammatory conditions. Our data show that the stimulatory effects of PRF on VEGF, BMP2, COX2, TNF-α, SPP1, and wound closure are not compromised by an inflammatory environment, which could explain why PRF is so clinically effective. PRF is already used in resective and regenerative periodontal surgery, for example for the treatment of intrabony defects. By showing that PRF stimulates wound healing–related molecules and cell migration even under inflammatory conditions, our study provides mechanistic support for its clinical application and underlines its value as a cost-effective autologous adjunct particularly in the surgical phase of periodontal therapy.

Periodontitis is a multifactorial inflammatory disease, involving a complex interplay of microbial, immunological, and environmental factors [51,52,53,54], which are difficult to imitate in their entirety in an in vitro model. To mimic inflammation in vitro, TNF-α was used in our experiments, as in previous studies by our group and other investigators [29,55], because it plays a central role in inflammation. It is one of the first cytokines to be released in an inflammatory reaction and represents a pleiotropic mediator with a wide variety of proinflammatory, autoimmune, malignant, and physiological functions [41,56]. TNF-α is mainly secreted by cells involved in the inflammatory process, such as granulocytes, macrophages and lymphocytes, but can also be released by local resident structural cells, such as endothelial cells and fibroblasts [41,57]. In periodontitis, TNF-α levels are increased in gingival crevicular fluid, saliva and serum [58,59]. Due to its relevance in the periodontal inflammatory process, we therefore decided to use this mediator [44]. In other in vitro studies, IL-1β, LPS, periodontal pathogens or biofilms were applied to simulate inflammation [11,60]. Future studies should investigate whether these proinflammatory and microbial factors, alone or in combination, at varying concentrations, impair the beneficial effects of PRF. Furthermore, we investigated a possible modulatory influence of inflammation in vitro. Future studies should clarify whether our observations can also be confirmed in animal models or clinically. It would also be interesting to know whether and to what extent hyperglycemia/diabetes, nicotine/smoking, and medications known to promote periodontitis modulate the effects of PRF. Vulnerable patients suffering from diabetes, rheumatoid arthritis, osteoporosis or other diseases could particularly benefit from the positive effects of PRF. In our study, PDL cells were used, as they are key players in periodontal destruction but also regeneration. However, the periodontium consists of multiple cell types such as gingival keratinocytes, fibroblasts, cementoblasts and osteoblast that can also contribute to the inflammatory and healing processes. Therefore, future studies should clarify whether our findings can also be observed in these cells.

## 5. Conclusions

This study explored the impact of PRF on critical markers of periodontal wound healing and tissue remodelling in PDL cells under normal and inflammatory conditions. PRF was found to induce the upregulation of VEGF, BMP2, COX2, TNF-α and SPP1, as well as stimulating wound closure in vitro. A proinflammatory stressor had no impact on the effects of PRF, suggesting that the beneficial actions of PRF may be fully preserved even in an inflammatory environment.

## Data Availability

The datasets presented in this article are not readily available because of ongoing studies but are available from the corresponding author on reasonable request.

## Abbreviations

The following abbreviations are used in this manuscript:

PRF	Platelet-rich fibrin
TNF-α	Tumor necrosis factor alpha
VEGF	Vascular endothelial growth factor
BMP2	Bone morphogenetic protein 2
COX2	Cyclooxygenase 2
GAPDH	Glyceraldehyde 3-phosphate dehydrogenase
PDL	Periodontal ligament
DMEM	Dulbecco’s Modified Eagle Medium
FBS	Fetal bovine serum
PBS	Phosphate-buffered saline
ELISA	Enzyme-linked immunosorbent assay
PCR	Polymerase chain reaction
cDNA	Complementary DNA
IL-1β	Interleukin 1 beta
LPS	Lipopolysaccharide
ANOVA	Analysis of variance
